# Novel *Naja atra* cardiotoxin 1 (CTX-1) derived antimicrobial peptides with broad spectrum activity

**DOI:** 10.1371/journal.pone.0190778

**Published:** 2018-01-24

**Authors:** Andrea Sala, Clotilde Silvia Cabassi, Davide Santospirito, Eugenia Polverini, Sara Flisi, Sandro Cavirani, Simone Taddei

**Affiliations:** 1 Department of Veterinary Science, University of Parma, Parma, Italy; 2 Department of Mathematical, Physical and Computer Sciences, University of Parma, Parma, Italy; nanyang technological university, SINGAPORE

## Abstract

*Naja atra* subsp. *atra* cardiotoxin 1 (CTX-1), produced by Chinese cobra snakes, belonging to *Elapidae* family, is included in the three-finger toxin family and exerts high cytotoxicity and antimicrobial activity too. Using as template mainly the tip and the subsequent β-strand of the first “finger” of this toxin, different sequences of 20 amino acids linear peptides have been designed in order to avoid toxic effects but to maintain or even strengthen the partial antimicrobial activity already seen for the complete toxin. As a result, the sequence NCP-0 (*Naja* Cardiotoxin Peptide-0) was designed as ancestor and subsequently 4 other variant sequences of NCP-0 were developed. These synthesized variant sequences have shown microbicidal activity towards a panel of reference and field strains of Gram-positive and Gram-negative bacteria. The sequence named NCP-3, and its variants NCP-3a and NCP-3b, have shown the best antimicrobial activity, together with low cytotoxicity against eukaryotic cells and low hemolytic activity. Bactericidal activity has been demonstrated by minimum bactericidal concentration (MBC) assay at values below 10 μg/ml for most of the tested bacterial strains. This potent antimicrobial activity was confirmed even for unicellular fungi *Candida albicans*, *Candida glabrata* and *Malassezia pachydermatis* (MBC 50–6.3 μg/ml), and against the fast-growing mycobacteria *Mycobacterium smegmatis* and *Mycobacterium fortuitum*. Moreover, NCP-3 has shown virucidal activity on Bovine Herpesvirus 1 (BoHV1) belonging to *Herpesviridae* family. The bactericidal activity is maintained even in a high salt concentration medium (125 and 250 mM NaCl) and phosphate buffer with 20% Mueller Hinton (MH) medium against *E*. *coli*, methicillin resistant *Staphylococcus aureus* (MRSA) and *Pseudomonas aeruginosa* reference strains. Considering these *in vitro* obtained data, the search for active sequences within proteins presenting an intrinsic microbicidal activity could provide a new way for discovering a large number of novel and promising antimicrobial peptides families.

## Introduction

The growing concern regarding increasing microbial antibiotic resistance is occurring worldwide [1,2]. Antibiotic resistance is often associated with marked morbidity and mortality in humans and animals and the number of resistant microorganisms is constantly growing [3,4]. Therefore, the development of novel antimicrobial therapies is urgently required [5,6].

Antimicrobial peptides (AMPs) possess a broad spectrum of antimicrobial activities against Gram-negative and Gram-positive bacteria, viruses, fungi and parasites [7].The main mechanism of action of AMPs against bacteria begins with an electrostatic interaction between cationic portions of antimicrobial peptides and negatively charged structures exposed on the surface of bacterial membranes. In Gram-negative bacteria the mechanism involves anionic phospholipids and LPS-associated phosphate groups exposed on the outer membrane surface. In Gram-positive bacteria, lacking outer membrane or LPS, AMPs are capable to interact with negatively charged teichoic and teichouronic acids of the cell envelope [8,9].

Following the initial membrane binding, peptides permeate the lipid bilayer by creating a toroid pore into the membrane or by using a carpet mechanism, leading to membrane damage and killing of the microorganism [10].

Several AMPs families do not directly alter membrane integrity but exhibit multiple mechanisms of action, targeting other conserved and essential components of the bacterial cells, in a process that provides them potent and more specific antimicrobial activities [8].

These important features in fighting microorganisms grant to AMPs a low potential to induce *de novo* resistance. Due to their prospective potency, rapid action, and broad spectrum of activity, antimicrobial cationic peptides have attracted attention as alternative or complementary antibiotics [11].

In nature, AMPs constitute a major component of the innate immune systems of most living organisms, including microorganisms, plants, invertebrates, and chordates and are divided in different families on the basis of amino acid sequences and structural characteristics [9,12,13]. These molecules can be found in many tissues and secretions of living organisms and some of them, like defensins or many α-helical AMPs, were also identified in animal venoms acting as toxins [14]. As other toxins found in venoms, these “cytotoxic” AMPs are the result of toxin recruitment events in which an ordinary protein gene is duplicated. The new gene is selectively expressed in the venom gland and is subject to frequent duplications, leading to functional and structural diversification of the secreted protein [15]. In some arthropod venoms cationic peptides synergistically work with neurotoxins to paralyze preys or deter aggressors. Once isolated from venom, these peptides exhibit, despite a certain degree of maintained cytotoxicity, common properties with AMPs including antimicrobial activity [14].

Snake venoms are complex mixtures of pharmacologically active peptides and proteins. Three-finger toxins (3-FTxs) belong to a superfamily of non-enzymatic proteins almost found in snakes belonging to *Elapidae* family. They are characterized by a three-fingered loop-folding topology dominated by β-sheet and can show different pharmacological activities, including haemolysis, cytotoxicity and muscles depolarization. Among the 3-FTxs, cardiotoxins (CTXs) and α-neurotoxins are the main toxic proteins from elapid snake venoms. [16,17]. Similarly to AMPs, these toxins can interact with anionic lipids or negatively charged oligosaccharides on cell membrane and subsequently form an oligomeric toxin complex. They can damage phospholipid bilayers through the formation of a membrane pore structure, and induce permeabilization both of the outer and the inner membrane [18–20]. Venoms could therefore be useful as possible source of new antimicrobial peptides. Moreover, since the amino acid sequence affects AMPs antimicrobial activity, the change of snake venoms cardiotoxins amino acid sequences could also improve their efficacy against several microorganism and/or reduce the cytopathic effect on eukaryotic cells [21].

The aim of this study is focused on the development and the characterization of novel antimicrobials peptides, designed starting from cardiotoxin 1 (CTX-1) of the Chinese cobra (*Naja atra atra*), in order to enhance its bactericidal activity and decrease the cytotoxicity showed by the ancestor against eukaryotic cells.

## Materials and methods

### a) NCPs design

Sequence generation: starting from the amino acid sequence of S-type cardiotoxin 1 (CTX-1) (accession number P60304) produced by the Chinese cobra (*Naja atra* subsp. *atra*), the sequences of novel 20 residues long AMPs were designed.

From the complete pro-protein sequence, the signal peptide has been previously detected using “SegnalP 4.1 Server” web server [22] and subsequently removed.

CTX-1 sequence was then scanned searching stretches suitable as cell-penetrating peptide (CPP). CPPs are short peptides (<50 amino acid) with the inherent ability of interacting and penetrating phospholipid membranes [23]. To identify CTX-1 sections of interest, the protein sequence have been scanned throughout its length using “CellPPD—Protein Scanning” web server in order to predict CPP fragments. The Support Vector Machine (SVM) based prediction model used was developed using a training set consisting in 708 peptides. SVM + motif based were the prediction mode selected because of the great accuracy achieved by this hybrid model, with an E-value cut off of 0.00001 and a SVM threshold of 0.0, and the possibility of selecting a window of 20 amino acid. The window length determines the length of fragments in which the protein has to be fragmented in a “sliding window” fashion. From the output, only 23 sequences predicted as CPP were considered amongst the 42 fragments obtained. These final candidates were sorted upon SVM score to get the predicted most potent CPP, obtaining the final candidate sequence [24].

**Fig 1:**
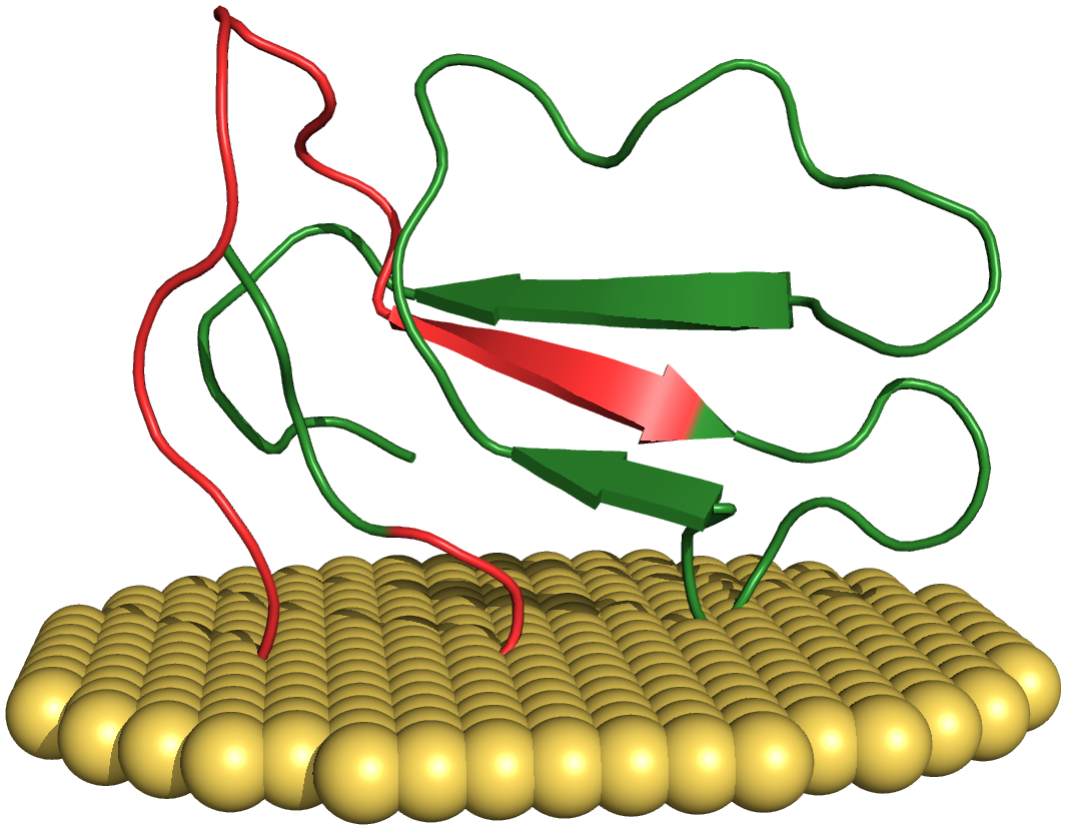
Interactions between CTXs and biologic membranes. Three-dimensional structure of the S-type CTX-1 (P60304) from *Naja atra* (Chinese cobra). The penetration of the membrane (colored in yellow) involves the tip of loop I only. The initial portion of the NCPs generating stretch (colored in red) appear mainly involved in this interaction. Visualized using the PyMOL Molecular Graphics System, Version 1.8 Schrödinger, LLC.

The final sequence KLIPIASKTCPAGKNLCYKM (Fig 1) has been therefore examined using the prediction tool of “CAMPR_3_” web server, to predict the antimicrobial activity of the submitted sequence by means of further four prediction models [25]:

- Support Vector Machine (SVM)
- Random Forests (RF)
- Artificial Neural Network (ANN)
- Discriminant Analysis (DA)

The submitted sequence was confirmed as potential AMP by all prediction models employed.

Subsequently, through single-residue substitutions, mutant sequences were rationally designed with the purpose of improving mainly the amphipathicity of the resulting peptides. These AMPs were named with the acronym NCP (*Naja* Cardiotoxin Peptide) followed by a progressive number. Tested NCPs are reported in Table 1. NCPs 3a and 3b are variants of NCP-3 obtained by replacing Trp4 with Phe4 and Leu4, respectively (Table 1).

**Table 1:** Amino acidic sequences of peptides belonging to the NCPs family.

Sequence ID	Primary Structure
Original CTX-1 stretch	KLIPIASKTCPAGKNLCYKM
NCP-0	KLIPIASKTCPAGKNLCYK**I**
NCP-2	KLIPILSKTIPAGKNLFYKI
NCP-3	KLIWILSKTIPAGKNLFYKI
NCP-3a	KLIFILSKTIPAGKNLFYKI
NCP-3b	KLILILSKTIPAGKNLFYKI

The substitutions in the amino acids composition, compared to the Original CTX-1 stretch, are marked in bold and underlined.

Each obtained sequence was validated through the prediction models reported above. Patterns of physico-chemical/structural characteristics of all these sequences (positive charge, hydrophobicity, amphipathicity, polar angle, etc.) were found consistent with those observed for AMPs.

**Fig 2:**
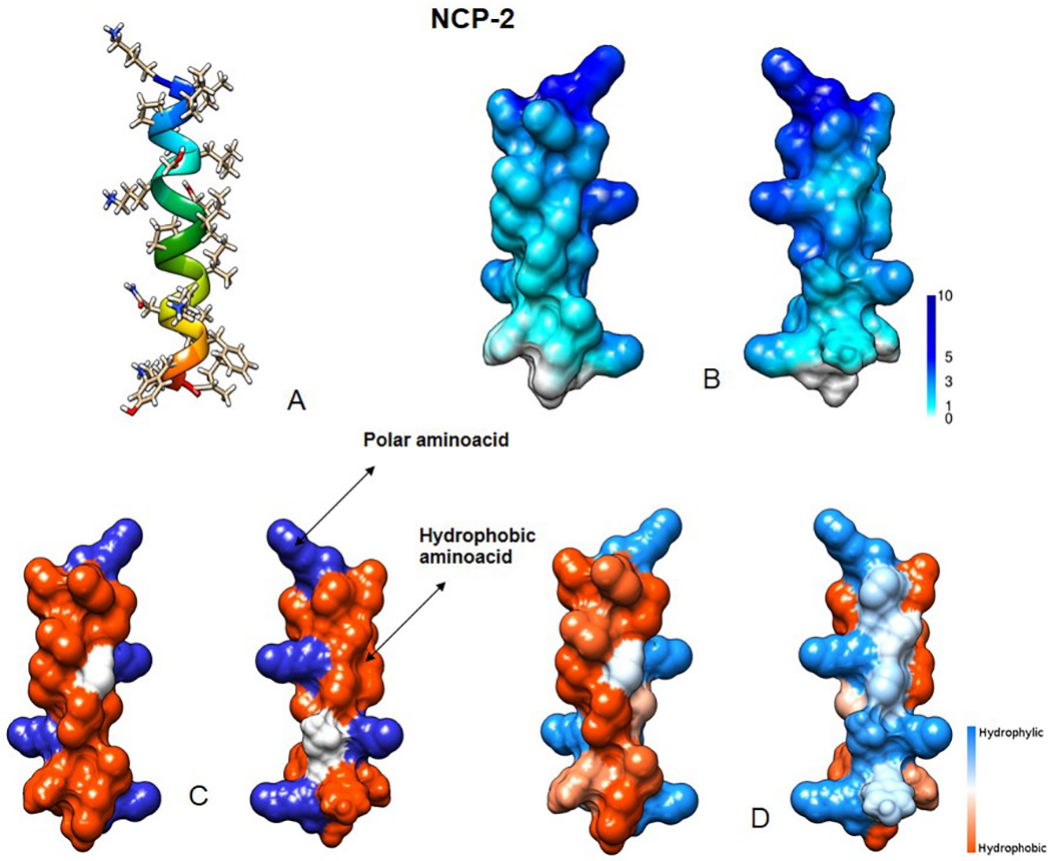
NCP-2 predicted structural features. (A) Three-dimensional structure. (B) Molecular surface colored by electrostatic potential (ranging from 1 to 10 kcal/(mol *e*)). (C) Distribution of basic (blue) and hydrophobic (red) amino acids. (D) Molecular surface colored by hydrophobicity (using the Kyte-Doolittle scale). Visualized using University of California, San Francisco (UCSF) Chimera.

**Fig 3:**
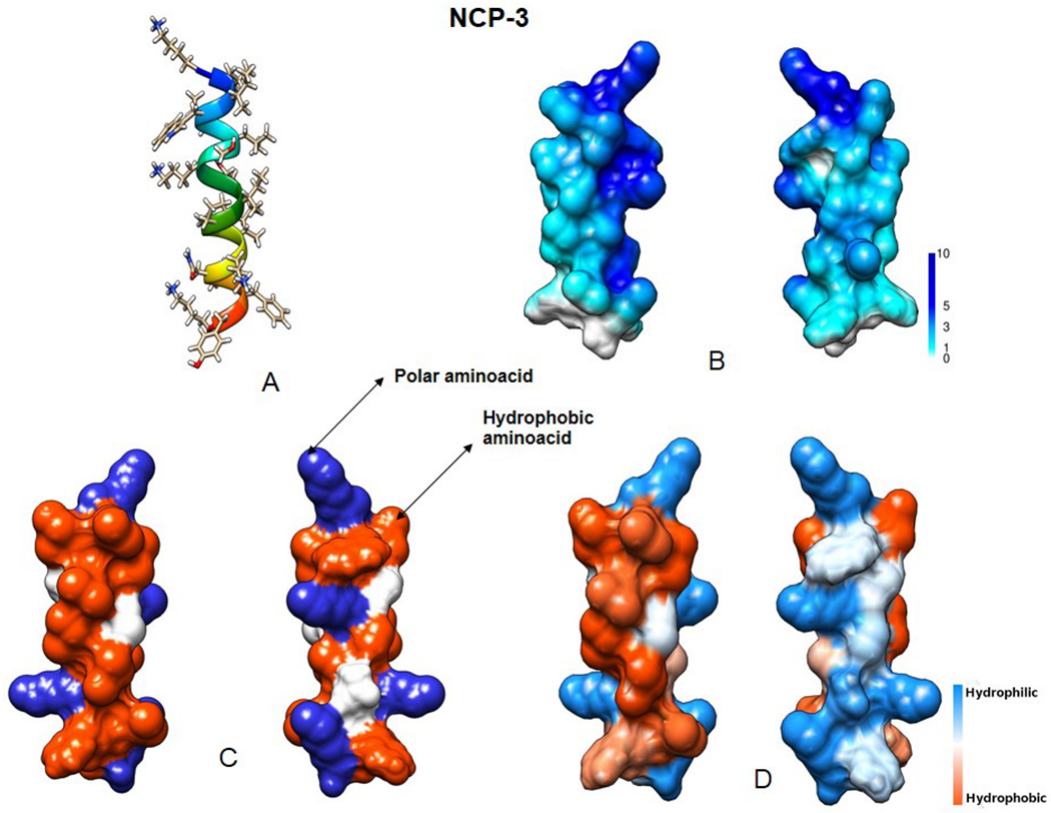
NCP-3 predicted structural features. (A) Three-dimensional structure. (B) Molecular surface colored by electrostatic potential (ranging from 1 to 10 kcal/(mol *e*)). (C) Distribution of basic (blue) and hydrophobic (red) amino acids. (D) Molecular surface colored by hydrophobicity (using the Kyte-Doolittle scale). Visualized using University of California, San Francisco (UCSF) Chimera.

Structural characteristics of our peptides were inferred through “The MCPep Server” web server [26]. In hydrophobic environment, they were all predicted as α-helical, stabilized by 15 interstrand hydrogen bonds, while in water only random coiled conformation was observed. The electrostatic potential of NCP-2 and NCP-3 revealed a surface with a large positive region due to the four Lys, and a large hydrophobic area on the opposite side of the molecule (Figs 2 and 3).

Peptides: NCP-0, NCP-2, NCP-3, NCP-3a and NCP-3b were synthesized from SelleckChem (Houston, TX, USA). Peptides purity (>90%), sequences and concentrations were assessed and provided by SelleckChem using High Pressure Liquid Chromatography (HPLC) and mass spectroscopy. The freeze-dried peptides were resuspended in a 10mM phosphate buffer (PB) solution (0.8709 g/L K_2_HPO_4_, 0.6804 g/L KH_2_PO_4_) at a stock concentration of 1 mg/ml. NCP-3a and NCP-3b, due to their predicted low water solubility, were previously diluted in 500 μl of DMSO and subsequently brought up to volume with PB.

Circular dichroism measurements: structural characterization of peptides, both in buffer solution and in vesicles, was made by means of circular dichroism (CD) measurements. In order to evaluate the peptide spatial structure when it comes in contact with bacterial membrane surfaces, an assay was performed onto Small Unilamellar Vesicles (SUV). Previous studies reported that *E*. *coli* membrane was constituted by phosphatidylethanolamine (DMPE) and phosphatidylglycerol (DMPG) at a molar ratio of approximately 75:25 [20]. Due to this, SUV were prepared as follows: phospholipids of DMPE and DMPG at a molar ratio of 75:25 were dissolved in chloroform/methanol (1:1, v/v) and then the solvent was evaporated using a Rotavapor system. It is worthwhile to note that also in *P*. *aeruginosa* membrane, PE and PG are the two most present kind of lipid (together with cardiolipin) and in about the same ratio [27]. Dried films were kept under N_2_ overnight and then hydrated in 10 mM phosphate buffer, pH 7.0. Afterwards, the lipids were sonicated with a Sonicator 3000 (Misonix) instrument, equipped with a micro-tip, for 10 min, until the lipid suspension becomes a transparent solution. To avoid the overheating of the sample, the sonication was performed at steps of 30 s, keeping the sample in a refrigerating bath. In the final solution with vesicles, the peptide: lipid molar ratio was 1:21. At the same time, to test the behaviour of NCP peptides in the presence of membranes mimicking the eukaryotic cell one, a second kind of SUV were prepared with an identical procedure, using phospholipids of dimyristoylphosphatidylcholine (DMPC) and cholesterol (Chol) at a molar ratio of 70:30 [28,29].

Circular dichroism spectra were recorded in the far-UV spectral region (250–185 nm) with a Jasco J715 spectropolarimeter, at a rate of 50 nm/min, using a cell of 0.1 cm optical path length at a peptide concentration of 0.2 mg/ml. Samples were thermostated at 20°C. A time constant of 4 s and a bandwidth of 2 nm were used. Spectra were recorded with a step of 0.2 nm and only one accumulation was necessary for peptides in buffer solution, while 5 accumulations for each spectrum were recorded for the samples in DMPE-DMPG vesicles, and 20 for the samples in DMPC-Chol vesicles, due to the strong absorbance of cholesterol under 200 nm.

The CD spectra were analyzed by means of the Dichroweb web server [30], using the CONTINLL algorithm [31] the reference data set number 7 [32] for the peptides in aqueous solution, and the SP175 or SMP180 [33], for the peptides in vesicles with the exception of NCP-2, for which the set 7 was ever used. The final choice of the algorithm and of the reference data set was made on the basis of the best NRMSD parameter [34].

### b) Antimicrobial activity

Gram positive and Gram negative strains: the following bacterial reference strains were used for this study: *Staphylococcus aureus* ATCC 25923, methicillin-resistant *Staphylococcus aureus* (MRSA) ATCC 43300, *Streptococcus agalactiae* ATCC 13813, *Escherichia coli* ATCC 25922, *Moraxella catarrhalis* ATCC 25238, *Pseudomonas aeruginosa* ATCC 27853, *Burkholderia cepacia* ATCC 17759, *Enterococcus hirae* ATCC 10541 and *Proteus mirabilis* ATCC 14153. In addition, field strains of human and animal origin were also tested: *Acinetobacter baumannii*, *Enterobacter cloacae* and *Klebsiella pneumonia*e subsp. *pneumoniae*. Animal strains were collected from patients referred to the Teaching Veterinary Hospital of the Department of Veterinary Science of the University of Parma. The human *Acinetobacter baumannii* strain was a multidrug-resistant strain belonging to our bacterial strain collection. The sources of each strain are reported in Table 2. Identification of field isolates was based on growth and colony characteristics, Gram staining, cellular morphology, catalase and oxidase reactions. Species identification was carried out using the API 20 E biochemical test system (bioMérieux, France), as well as conventional biochemical tests [35].

**Table 2:** MBC values for NCP-0, NCP-2,NCP-3, NCP-3a and NCP-3b against Gram-negative and Gram-positive bacteria.

	NCP-0	NCP-2	NCP-3	NCP-3a	NCP-3b
*Escherichia coli* ATCC 25922	>100 μg/ml	12.5 μg/ml	12.5 μg/ml	6.3 μg/ml	12.5 μg/ml
*Pseudomonas aeruginosa* ATCC 27853	>100 μg/ml	12.5 μg/m	12.5 μg/ml	25 μg/ml	25 μg/ml
*Acinetobacter baumannii* (herpetological, cloacal)	>100 μg/ml	1.6 μg/ml	1.6 μg/ml	ND^a^	ND
*Acinetobacter baumannii* (herpetological, cloacal)	>100 μg/ml	1.6 μg/ml	1.6 μg/ml	ND	ND
*Acinetobacter baumannii* (ornithological, cloacal)	>100 μg/ml	50 μg/ml	6.3 μg/ml	25 μg/ml	25 μg/ml
*Acinetobacter baumannii* (human, urinary)	>100 μg/ml	1.6 μg/ml	1.6 μg/ml	25 μg/ml	25 μg/ml
*Klebsiella pneumoniae* subsp. *pneumoniae* (herpetological, cloacal)	>100 μg/ml	>100 μg/ml	50 μg/ml	50 μg/ml	25 μg/ml
*Enterobacter cloacae* (herpetological, cloacal)	>100 μg/ml	>100 μg/ml	25 μg/ml	ND	ND
*Burkholderia cepacia* ATCC 17759	50 μg/ml	>100 μg/ml	50 μg/ml	50 μg/ml	25 μg/ml
*Proteus mirabilis* ATCC 14153	>100 μg/ml	>100 μg/ml	>100 μg/ml	>100 μg/ml	>100 μg/ml
*Moraxella catarrhalis* ATCC 25238	1.6 μg/ml	1.6 μg/ml	1.6 μg/ml	3.1 μg/ml	12.5 μg/ml
*Methicillin-resistant Staphylococcus aureus ATCC 43300*	>100 μg/ml	>100 μg/ml	6.3 μg/ml	6.3 μg/ml	6.3 μg/ml
*Staphylococcus aureus ATCC 22953*	>100 μg/ml	50 μg/ml	1.6 μg/ml	12.5 μg/ml	50 μg/ml
*Enterococcus hirae ATCC 10541*	>100 μg/ml	>100 μg/ml	1.6 μg/ml	6.3 μg/ml	12.5 μg/ml
*Streptococcus agalactiae ATCC 13813*	>100 μg/ml	12.5 μg/ml	1.6 μg/ml	3.1 μg/ml	12.5 μg/ml

^a^ND: not determined

MBC evaluation: minimal bactericidal concentration (MBC) was considered as the ability of peptides to kill 99.9% of bacteria. It was evaluated by broth microdilution assay, following CLSI guidelines [36]. MBC assay was performed in 96 wells microtiter plates by incubating peptides in PB at concentrations ranging from 0.1 μg/ml to 100 μg/ml with 5x10^5^ CFU/ml of bacterial suspension in a final volume of 100 μl. Bacterial suspension was prepared as previously described [37] and inoculated within 30 min. Growth and sterility controls were performed. Bacterial suspensions and peptides were incubated for 2 hours at 37°C. Then, 20 μl of each dilution were plated onto adequate agar media and incubated for 24 h at 37°C for the CFU count.

Time kill assay: antibacterial activity over time was evaluated by broth microdilution assay [37]. In particular, the standardized bacterial suspension was exposed to peptides at the concentration of 12.5 μg/ml. Twenty microliters aliquots were withdrawn at fixed intervals (5, 10, 15, 30, 60 and 120 min) and spread onto adequate solid culture medium. After 24 hours of incubation at 37°C, CFU were counted. Controls were run without peptide and in the presence of peptide solvent.

Antimycobacterial activity: anti-mycobacterial activity of NCP-3 was evaluated on *Mycobacterium fortuitum* DSMZ 46621 (ATCC 6841) and *Mycobacterium smegmatis* DSMZ 43756 (ATCC 19420) by resazurin (7-Hydroxy-3H-phenoxazin-3-one 10-oxide) assay performed in microtiter plates, in presence and in absence of 250 μM EDTA. NCP-3 was diluted in PB and tested at the concentration of 100 μg/ml. The final volume contained 50% of Middlebrook 7H9 broth when the peptide was tested in presence of EDTA or 25% of Middlebrook 7H9 broth when tested in absence of EDTA. Growth and sterility controls were performed. Inocula were prepared as follows: mycobacteria were incubated in Middlebrook 7H9 broth at 37°C for 24 hours and then passed through a 25G needle 5–10 times, to break up cells clusters. The turbidity of the bacterial suspension was immediately measured by a spectrophotometer. A 600 nm absorbance in the range 0.08–0.13 was considered to correspond to a bacterial concentration of 10^8^ CFU/ml. The 10^8^ CFU/ml bacterial suspension was further diluted 1:100 to obtain a bacterial concentration of about 10^6^ CFU/ml. Within 15 min, 100 μl of the suspension 10^6^ CFU/ml were inoculated in each well, obtaining a final bacterial concentration of about 5x10^5^ CFU/ml. After bacteria inoculation, microtiter plates were incubated at 37°C for 72 hours. Then, 20 μl of 0.01% resazurin were added to each well. After further 24 hours of incubation at 37°C, bacterial viability was determined by evaluating the conversion of resazurin (blue) to resorufin (pink).

Antifungal activity: antifungal activity was also evaluated on unicellular mycetes *Candida albicans* ATCC 10231, *Candida glabrata* ATCC 90030 (DSMZ 11226) and *Malassezia pachydermatis* DSMZ 6172. Fungi were cultivated in Sabouraud dextrose agar for 24–48 hours at 35°C. The inoculum was prepared by suspending in PB five colonies of about 1 mm diameter. The suspension was adjusted to a final optical density of 0.5 McFarland standard (1-5x10^6^ cells/ml). Fifty microliters of each peptide solutions ranging from 0.1 μg/ml to 100 μg/ml were added in 96 wells microtiter plates with 50 μl of fungal inoculum. Growth and sterility controls were performed. After 2 hours of incubation at 35°C, aliquots of 20 μl were withdrawn from each well and spread on Sabouraud dextrose agar. After 24 hours of incubation at 35°C, CFU were counted.

Antiviral activity: Antiviral activity of NCP-3 was evaluated against Bovine Herpesvirus-1 (BoHV-1) by virus neutralization assay, performed as follows. Twenty-five microliters of 400 μg/ml NCP-3 were added to the first line of wells of 96-well plates. Twenty-five microliters of minimum essential medium (MEM) were added to each well of the plates and serial twofold dilutions were performed. Positive and negative controls were included. Twenty-five microliters of MEM containing 100 TCID50 of BoHV-1 were added to each well. Therefore, after viral inoculum addition, NCP-3 concentrations ranged from 100 μg/ml to 3.1 μg/ml. After 2 h of incubation at room temperature, 50 μl of a 2x10^5^ cell/ml Madin Darby Bovine Kidney [(MDBK) ATCC, CCL-22] [38] cell suspension, in MEM containing 10% of fetal bovine serum, were added to each well and plates were incubated for 3 days at 37°C in a humidified atmosphere of 95% air and 5% CO_2_. The expression of viral infectivity and NCP-3 virus neutralizing activity were evaluated by phase contrast microscopy through detection of cytopathic effect.

Antimicrobial activity in presence of NaCl: antimicrobial activity of peptides at 12.5 μg/ml and 100 μg/ml concentrations was tested on *E*. *coli* ATCC 25922, *P*. *aeruginosa* ATCC 27853 and methicillin-resistant *S*. *aureus* ATCC 43300 in presence of increasing salt concentrations (125 and 250 mM NaCl). Bacterial suspension and peptides were incubated for 2 hours at 37°C and then plated for CFU counts.

Antimicrobial activity in presence of 20% of Mueller-Hinton broth: fifty microliters of peptides, at 12.5 μg/ml and 100 μg/ml in PB, were added to microtiter plate wells containing 20% of Mueller-Hinton broth and 5x10^5^ CFU/ml of one of the following bacteria: *E*. *coli* ATCC 25922, *P*. *aeruginosa* ATCC 27853 and methicillin-resistant *S*. *aureus* ATCC 43300. After 2 hours of incubation at 37°C, bacteria were plated onto solid culture medium and after further 24 hours at 37°C CFU were counted. Growth and sterility controls were performed.

### c) Mechanism of action

Permeabilization of the bacterial internal membrane by propidium iodide (PI) assay: the dead-cell stain was performed as previously described [39,40] on *P*. *aeruginosa* ATCC 27853 in presence of 12.5 μg/ml of NCP-3. Negative and positive controls were obtained in the absence of peptides and in the presence of 1 mM EDTA and 0.5% Triton X-100, respectively.

Permeabilization assay: the permeabilization of bacterial membranes caused by 12.5 μg/ml of NCP-2 or NCP-3 was evaluated on *E*. *coli* ML-35pYC, as previously described [37]. Bacteria were inoculated in 15 ml of MH broth containing 50 μg/ml ampicillin and incubated overnight at 37°C. Three hundred microliters of bacterial suspension were added to 15 ml of fresh MH broth containing 50 μg/ml ampicillin and incubated for 2–3 h at 37°C. The suspension was then centrifuged at 1000 g for 10 min and the pellet resuspended in PB. Sixty microliters of a 1.5 mM CENTA or a 15 mM ONPG solution, freshly prepared in PB, were added to 60 μl of bacterial suspension and placed in a cuvette containing 480 μl of PB. Peptide was added and the kinetic of color formation was followed for 60 min at 405 nm (CENTA) and 600 nm (ONPG).

### 4) Cytotoxicity assessment

Haemolysis test: freshly withdrawn sheep heparinized red blood cells (RBCs) were centrifuged at 100 g for 15 min and washed three times with PBS, centrifuged at 1000 g for 10 min and resuspended to concentration of 2% (v/v) in PB containing 308 mM sucrose to maintain cell osmolarity. Fifty microliters of RBCs were incubated with 50 μl of different peptide concentrations for 1 hour at room temperature. The suspension was then centrifuged at 1000 g for 5 min and haemolysis measured at 450 nm. Negative controls (0% haemoglobin release), obtained in absence of peptides, and positive controls (100% haemoglobin release), obtained in presence of 1% Tween 20, were carried out. Haemolysis percentage was calculated as follows:

$$\left(1-\frac{A_{pep}-A_{PB}}{A_{tween}-A_{PB}}\right)\times 100$$

where A_pep_ represents the optical density of the sample at 450 nm, A_tween_ the optical density of the positive control and A_PB_ the optical density of the negative control.

Cytotoxicity test: peptides cytotoxicity was evaluated on MDBK cells. About 5x10^5^ cells/ml were incubated overnight at 37°C in a humidified atmosphere of 95% air and 5% CO_2_ in presence of 100 μg/ml of peptides. To assess cells vitality, 20 μl of Trypan Blue were added to each well and plates were incubated 5 min at 37°C. Negative controls were performed in absence of peptide and positive controls in presence of 0.2% Triton X-100.

## Results

### a) NCPs design

Circular dichroism (CD) measurements: CD spectra were analyzed by means of the CONTILL algorithm of the Dichroweb server [30] using the set 7 as the best reference data set, basing on the calculated NRMSD value (see also the Materials and methods section). However, it should be underlined that the obtained secondary structure percentages are only indicative of a predominance of a certain kind of secondary structure, due to the fact that the databases available are not completely suitable for such short peptides [30], and that the contribution to the spectra both of the aromatic amino acids (15% of the total number of residues in NCP3) and of structural irregularities can vitiate the CD signal [41].

**Fig 4:**
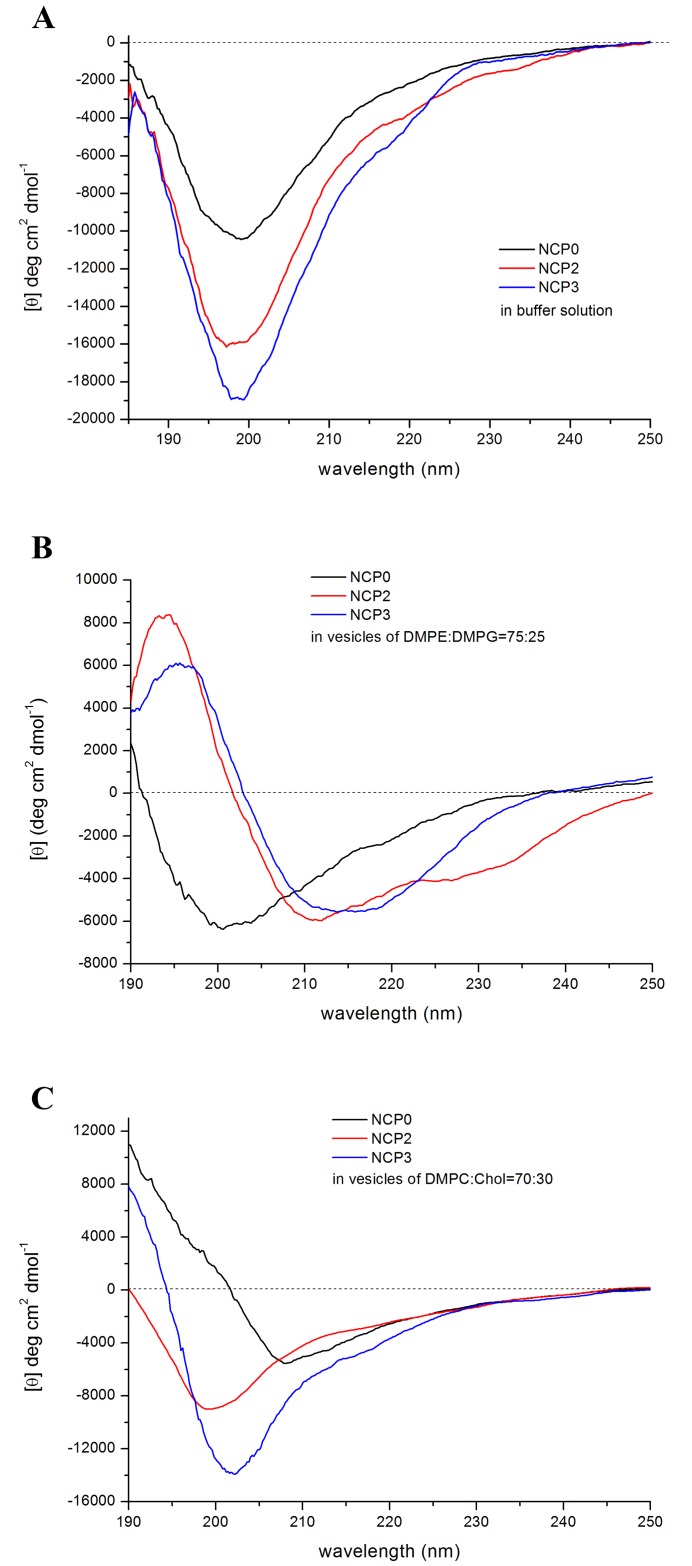
CD spectra of NCPs. (A) CD spectra of NCP-0, NCP-2 and NCP-3 in 10 mM phosphate buffer solution at pH 7. (B) Spectra of the same peptides recorded in the presence of DMPE-DMPG (75:25) vesicles at a peptide:lipid molar ratio of 1:21. (C) Spectra of the same peptides recorded in the presence of DMPC-Chol (70:30) vesicles at at a peptide:lipid molar ratio of 1:21.

The CD spectra of NCP-0, NCP-2 and NCP-3 in 10 mM phosphate buffer solution at pH 7 are shown in Fig 4A. All the spectra present a typical random coil profile. The analysis reported an overall content of unordered secondary structure that is 67% for NCP-0, 72% for NCP-2 and 67% for NCP-3. NCP-0 spectrum, which shows a lower intensity than the other two, and NCP-3 one, which has a very weak and large shoulder around 220 nm, indicate the presence of small amounts of ordered secondary structure in respect with NCP-2, mainly due to turns and beta-type structures. It was not possible to record the CD spectra of NCP-3a and NCP-3b, due to the high scattering of the solutions at the wavelengths used.

The CD spectra of NCP peptides were then recorded in the presence of DMPE-DMPG mixed vesicles—that mimic bacterial membrane—at a peptide:lipid molar ratio of 1:21. The spectra showed a very different behavior compared to the ones in solution (Fig 4B). The spectrum of NCP-0 indicates the persistence of a high amount of unordered structure (45%), even if the broadening of the negative band and its slight shift to 200/202 nm could suggest the presence of a beta-like secondary structure, as indeed was revealed by the spectrum analysis (36%). The NCP-2 and NCP-3 peptides with vesicles, instead, adopted a different ordered structure. NCP-2 shows a helix-like profile, in which, however, the slight red shift of the negative bands, compared to the canonical alpha helix spectrum, could indicate the presence of a beta structure too. Furthermore, the less intense negative band at 225 nm could suggest the presence of a 3_10_ helical component. The CD analysis algorithm reported a 20% of alpha helix (vs 4% in buffer solution) but also an increase of the beta content (from 13% in buffer to 25% in vesicles), while it was not able to extract the 3_10_ helix component. Indeed, the calculated spectra fit less well with the experimental one in the range of 220–230 nm. The unordered structure amount was 35%.

NCP-3 has the typical spectrum of a beta sheet secondary structure, with a broad band around 215 nm and a positive peak at about 197 nm. The analysis indicated a 43% of beta structure and a 35% of unordered one. Alpha and beta structures are both typical of AMPs and clearly indicate a strong membrane interaction.

To investigate the structural behavior of NCP peptides in the presence of model membranes that mimic the eukaryotic cell one, CD spectra in the presence of neutral DMPC-Chol vesicles were also recorded (Fig 4C). The NCP-2 peptide shows in this environment a random coil profile, with a calculated unordered structure of 62%. In NCP-3 the spectrum resembles to the one in buffer solution, with its weak and large shoulder around 220 nm, but the slight shift of the negative band from 197 nm in buffer to 202 nm in DMPC-Chol reveals an increase of beta secondary structure to 38%, even if the unordered one still remain high (45%). The NCP-0 peptide, differently from what happens in DMPE-DMPG vesicles, assumes a relevant amount of ordered structure, highlighted by the broad negative band with a minimum at 208 nm. In fact, the calculated alfa and beta structures percentage are of 18% and 34%, respectively, while the unordered one is only 35%.

Again, for both kind of vesicles, the CD spectra of the two peptides NCP-3a and NCP-3b show a very high scattering that prevent the signal recording also in the presence of vesicles.

### b) Antimicrobial activity

MBC evaluation: MBC values for the tested peptides against Gram-negative and Gram-positive bacteria are reported in Table 2. None or only slight activity was observed against almost all tested bacteria for NCP-0 (reference peptide). In general, NCP-2 showed a better activity against Gram-negative bacteria, than against Gram-positive ones. NCP-2 was particularly active against *P*. *aeruginosa* ATCC 27853 (MBC = 12.5 μg/ml), and also very effective against 3 out of 4 *A*. *baumannii* field strains (MBC = 1.6 μg/ml). Among Gram-positive bacteria, only *S*. *agalactiae* have shown sensitivity to NCP-2 (MBC = 12.5 μg/ml).

NCP-3 showed higher or at least equal antimicrobial activity compared with the other peptides. Equally to NCP-2, NCP-3 exhibited excellent activity against all *A*. *baumannii* field strains (MBC = 1.6 μg/ml for 3 of 4 bacterial strains) and performed even better than NCP-2 against *P*. *aeruginosa* ATCC 27853 (MBC = 6.3 μg/ml). In addition, NCP-3 activity against Gram-positive bacteria was excellent, with MBCs ranging from 1.6 μg/ml to 6.3 μg/ml. Great sensitivity was shown by *M*. *catarrhalis* ATCC 25238 against all NCPs tested.

**Fig 5:**
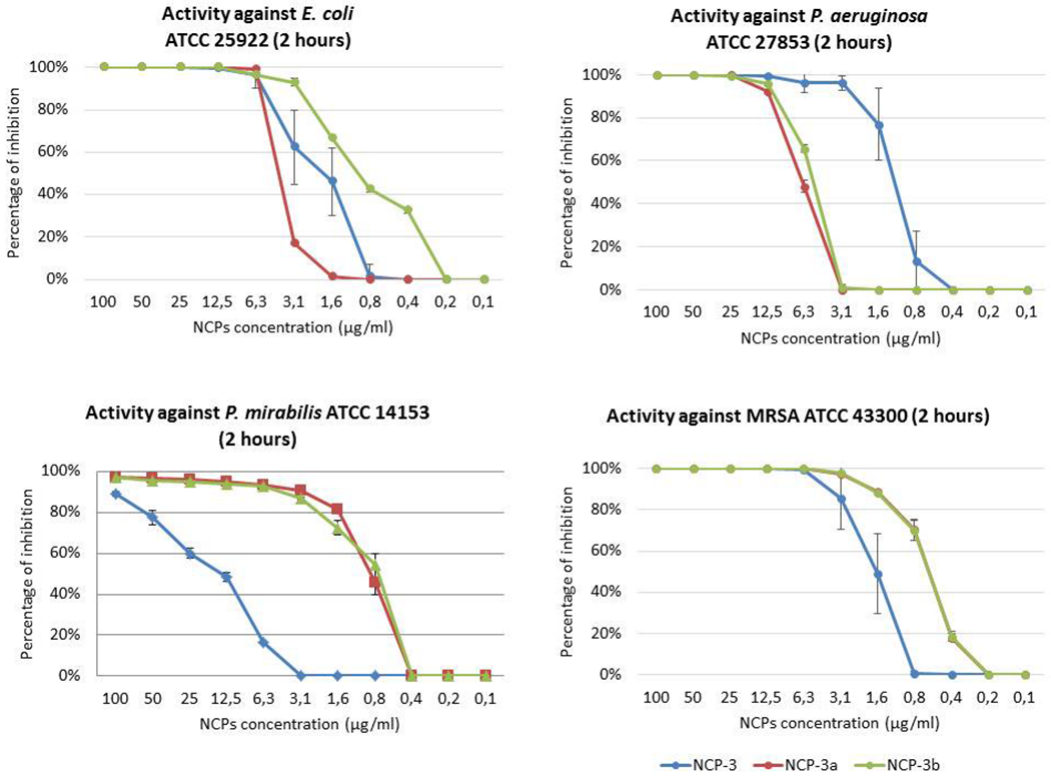
Activity of NCP-3, NCP-3a and NCP-3b against *E*. *coli* ATCC 25922, MRSA ATCC 43300, *P*. *mirabilis* ATCC 14153 and *P*. *aeruginosa* ATCC 27853. Increasing concentrations of peptides were incubated for 2 hours with 5x10^5^ CFU/ml of bacterial suspension. Percentage of inhibition was evaluated by CFU count.

NCP-3 variants, NCP-3a and NCP-3b, showed good activity against Gram-positive bacteria. The two variants showed a better antimicrobial activity compared to NCP-0 and generally also to NCP-2 (Table 2 and Fig 5).

While NCP-3 showed the best activity against *P*. *aeruginosa* ATCC 27853, the substitution at amino acid position 4 of Trp with Phe (NCP-3a) improved the antimicrobial activity against MRSA ATCC 43300. On the other hand, the substitution at the same amino acid position of Trp with Leu (NCP-3b) improved the antimicrobial activity against *E*. *coli* ATCC 25922. Moreover, differently from NCP-3, both NCP-3a and NCP-3b exhibited a strong activity against *P*. *mirabilis* ATCC 14153.

**Fig 6:**
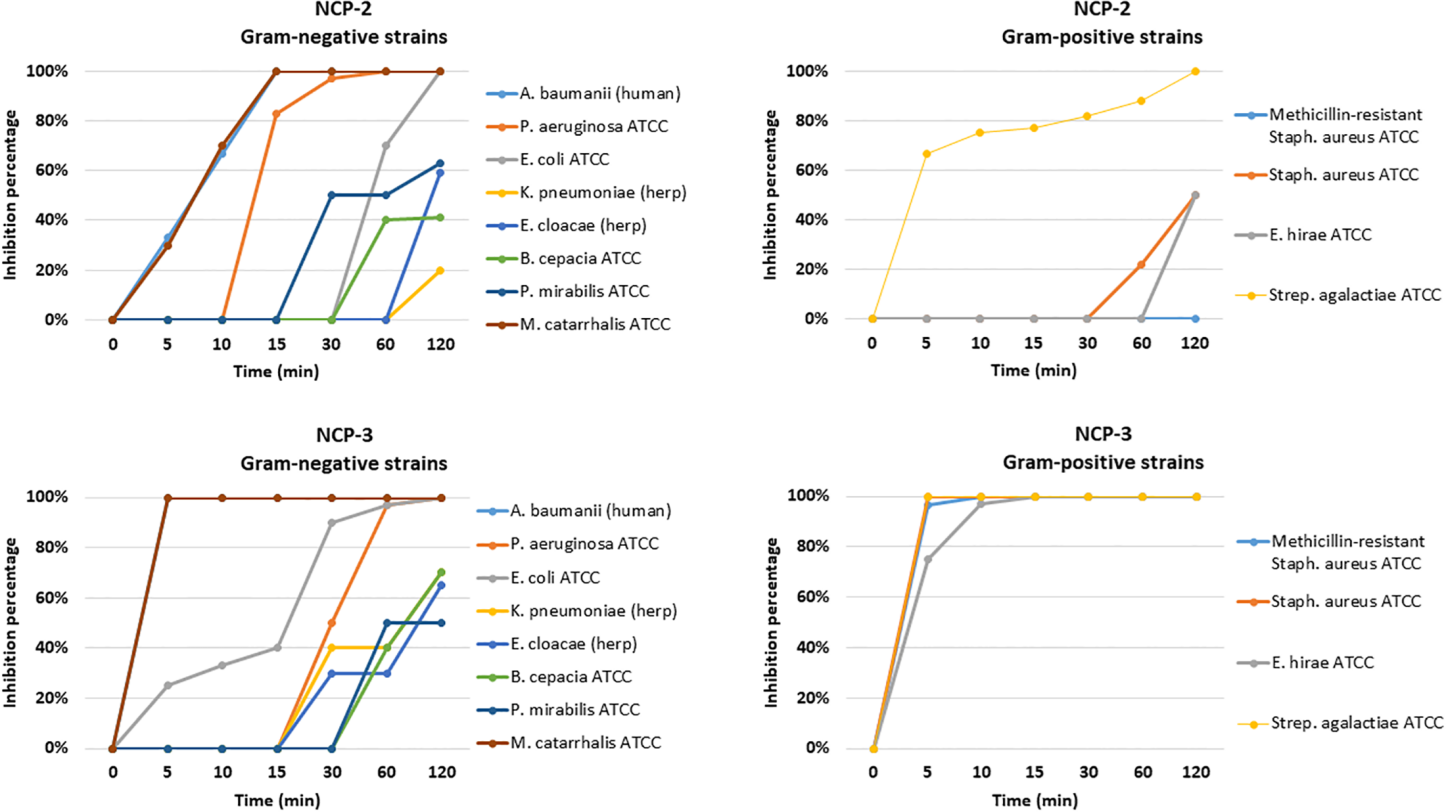
Time kill assay for NCP-2 and NCP-3 against Gram-negative and Gram-positive bacteria. Bacterial suspensions (5x10^5^ CFU/ml) were exposed to peptides at the concentration of 12.5 μg/ml. Percentage of inhibition was evaluated at fixed time intervals by CFU count.

Time kill assay: The results of the time-kill assay are reported in Fig 6. NCP-2 and NCP-3 confirmed an excellent antimicrobial activity against *A*. *baumannii* (human) and *M*. *catarrhalis* ATCC 25238, reaching 100% of inhibition within 15 min for NCP-2 and 5 min for NCP-3. Regarding Gram positive bacteria, NCP-3 reached 100% of inhibition within 15 min for all the tested strains and NCP-2 showed a good antimicrobial activity against *Streptococcus agalactiae* ATCC 13813.

**Fig 7:**
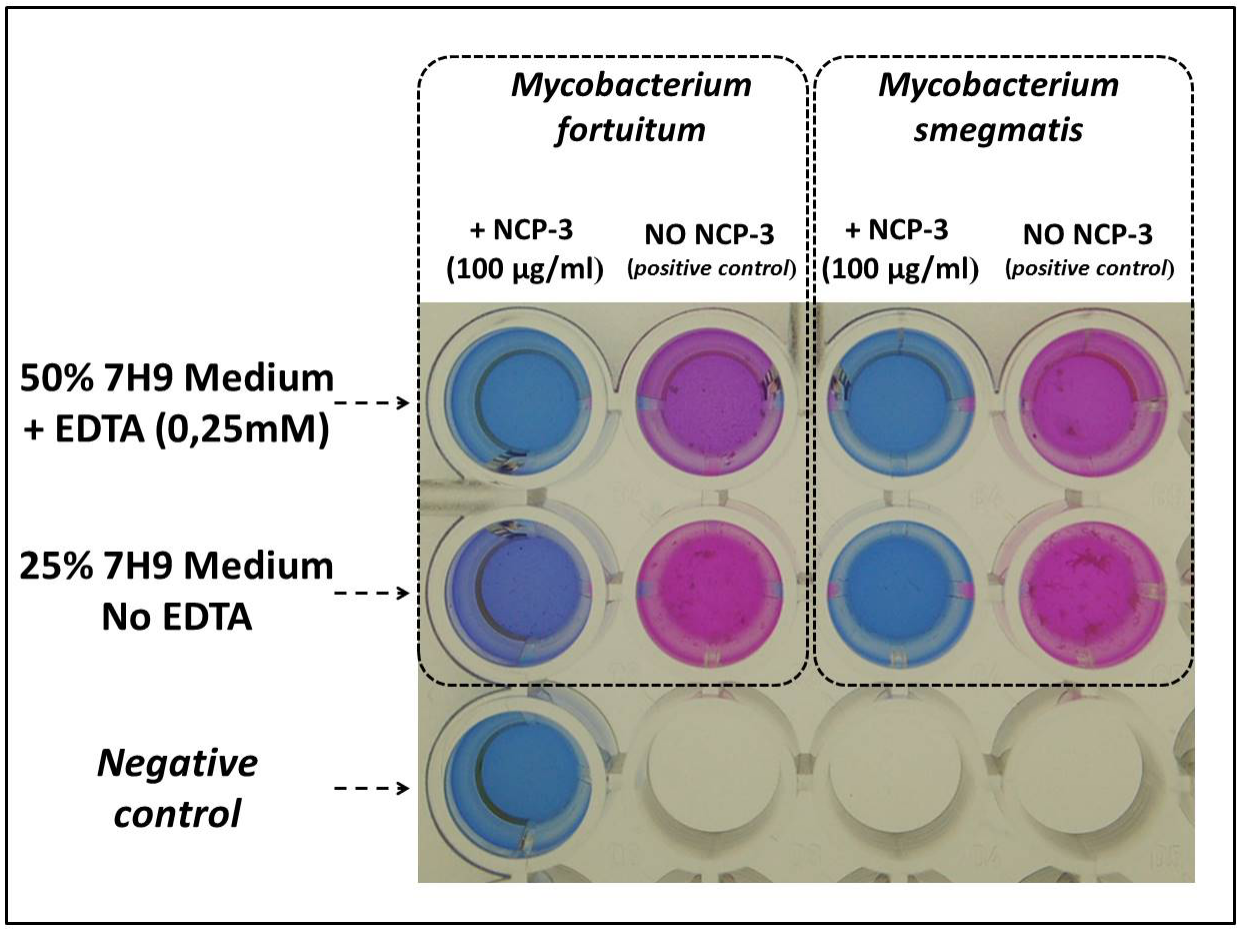
Representative results of the anti-mycobacterial activity of NCP-3 against *Mycobacterium fortuitum* DSMZ 46621 and *Mycobacterium smegmatis* DSMZ 43756 evaluated by resazurin assay. Bacterial suspensions (5x10^5^ CFU/ml) were exposed to NCP-3 at 37°C for 72 hours, then resazurin was added and plates were read after 24 hours at 37°C. Viable cells convert resazurin (blue) to resorufin (pink).

Anti-mycobacterial activity: The results concerning the antimicrobial activity of NCP-3 against mycobacteria are reported in Fig 7. NCP-3 at a concentration of 100 μg/ml completely inhibited resazurin conversion, due to *Mycobacterium fortuitum* DSMZ 46621 and *Mycobacterium smegmatis* DSMZ 43756 growth, in presence of EDTA. In absence of EDTA, *Mycobacterium smegmatis* DSMZ 43756 growth was completely inhibited, whilst the presence of an incomplete resazurin conversion indicated a limited growth of *Mycobacterium fortuitum* DSMZ 46621.

Antifungal activity: The results of antifungal activities are reported in Table 3. NCP-2 showed a good activity against *M*. *pachydermatis* DSMZ 6172, while higher concentration are required to be effective towards *C*. *albicans* ATCC 10231 and *C*. *glabrata* ATCC 90030 (DSMZ 11226). NCP-3 showed a good activity towards *C*. *albicans* ATCC 10231 and *M*. *pachydermatis* DSMZ 6172, while it was less effective against *C*. *glabrata* ATCC 90030. NCP-3a was the most effective peptide against all the tested fungal strains.

**Table 3:** MBC values for NCP-0, NCP-2, NCP-3, NCP-3a and NCP-3b against fungal strains.

	NCP-0	NCP-2	NCP-3	NCP-3a	NCP-3b
*Candida albicans* ATCC 10231	>100 μg/ml	50 μg/ml	12.5 μg/ml	6.3 μg/ml	12.5 μg/ml
*Candida glabrata* ATCC 90030	>100 μg/ml	>100 μg/ml	50 μg/ml	25 μg/ml	100 μg/ml
*Malassezia pachydermatis* DSMZ 6172	>100 μg/ml	6.3 μg/ml	6.3 μg/ml	6.3 μg/ml	25 μg/ml

Antiviral activity: NCP-3, at the concentration of 25 μg/ml, completely inhibited the viral activity of BoHV-1 on MDBK cells.

Antimicrobial activity in presence of NaCl or MH broth: Inhibition percentages of bacterial growth due to the peptides in presence of 125 and 250 mM NaCl or 20% of MH broth are reported in Table 4. NCP-0 did not showed any antimicrobial activity in the tested conditions. NCP-2, as expected, showed antimicrobial activity only against Gram-negative bacteria tested, while NCP-3 maintained the antimicrobial activity in all the tested media and against all the tested strains.

**Table 4:** Antimicrobial activity (inhibition percentages) of NCP-0, NCP-2 and NCP-3 in presence of sodium chloride or MH broth.

			*Escherichia coli* ATCC 25922		*Pseudomonas aeruginosa* ATCC 27853		*Methicillin-resistant Staphylococcus aureus* ATCC 43300	
Peptide concentration (μg/ml)			100	12.5	100	12.5	100	12.5
**NCP-0**	PB		3%	0%	2%	0%	2%	0%
	NaCl	125 mM	0%	0%	0%	0%	0%	0%
		250 mM	0%	0%	0%	0%	0%	0%
	MH broth	20%	0%	0%	0%	0%	0%	0%
**NCP-2**	PB		100%	100%	100%	100%	0%	0%
	NaCl	125 mM	70%	51%	90%	86%	0%	0%
		250 mM	68%	43%	78%	65%	0%	0%
	MH broth	20%	100%	100%	87%	0%	0%	0%
**NCP-3**	PB		100%	100%	100%	100%	100%	100%
	NaCl	125 mM	100%	100%	100%	100%	100%	97%
		250 mM	100%	97%	100%	100%	100%	38%
	MH broth	20%	100%	100%	100%	100%	100%	97%

### b) Mechanism of action

**Fig 8:**
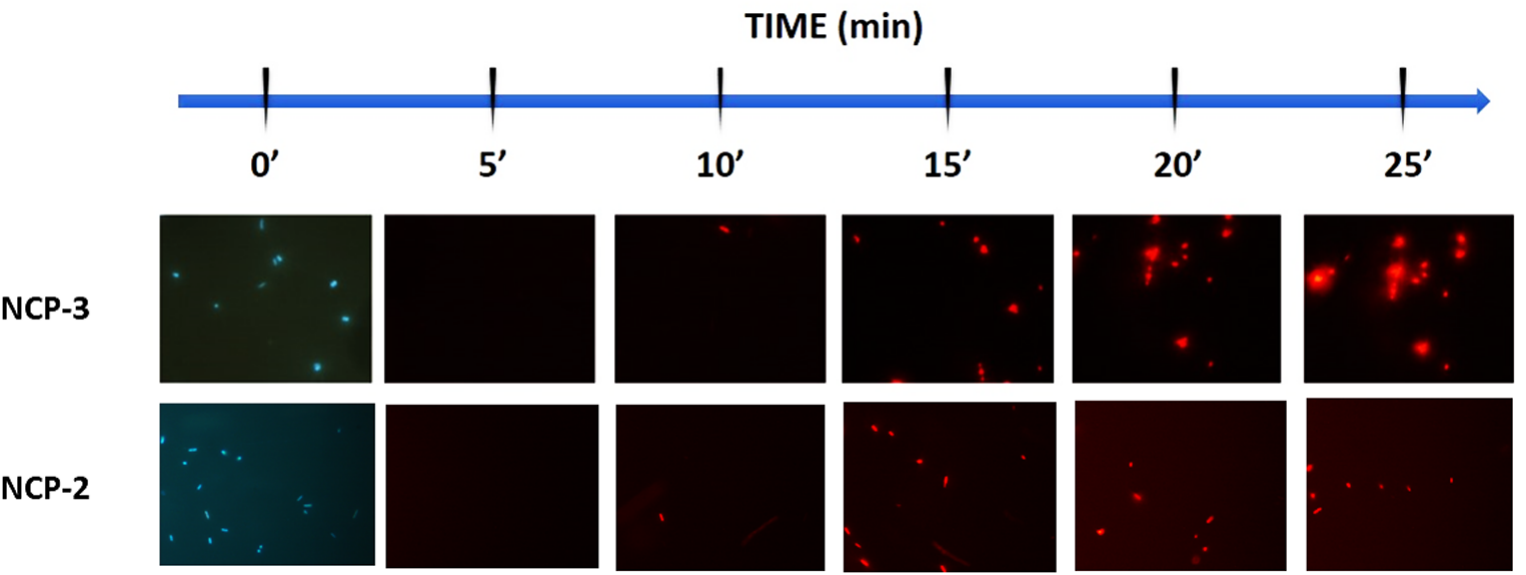
Propidium iodide (PI) dead-cell stain assay. Permeabilization of the inner membrane of *P*. *aeruginosa* ATCC 27853 as a function of contact time (min) with 12.5 μg/ml of NCP-3 and NCP-2. Viable cells are impermeable to PI and are blue due to counterstaining with 4’,6-diamidino-2-phenylindole (DAPI). Conversely, PI penetrates and stains the DNA of membrane-compromised cells (red emission).

Permeabilization of the bacterial internal membrane by PI assay: The stain-dead assay highlighted fast permeabilization of the inner membrane, with the initial occurrence of red fluorescence within 15 min after contact with NCP-2 or NCP-3 (Fig 8).

**Fig 9:**
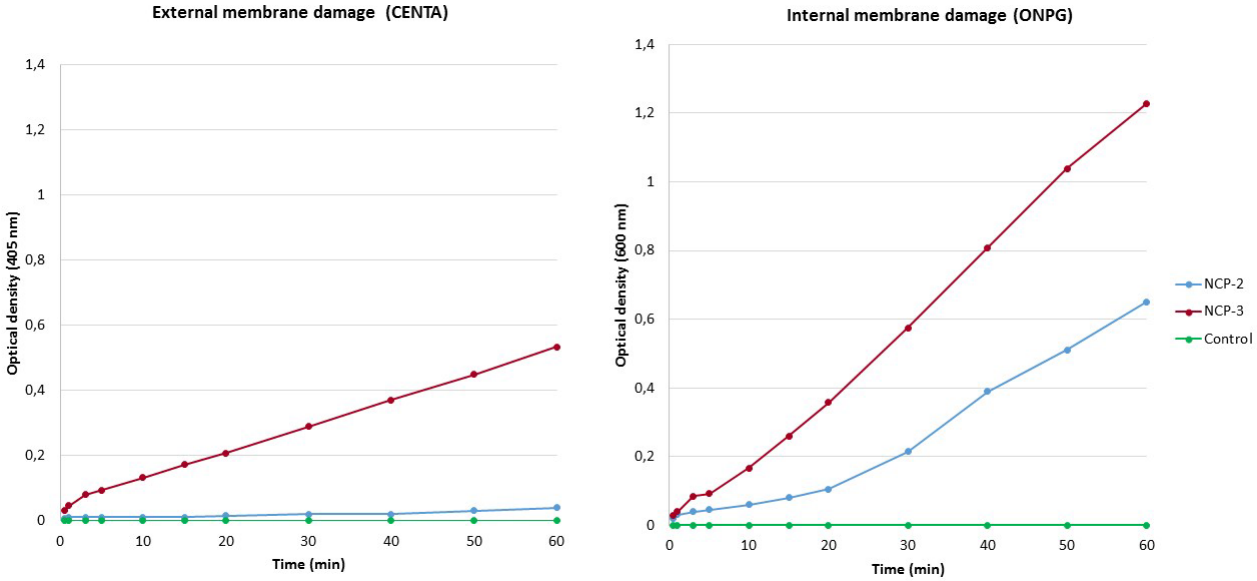
Permeabilization of bacterial membranes assay. NCP-2 and NCP-3-mediated membrane permeabilization of *E*. *coli* ML-35 pYC. Inner membrane damage was evaluated at 600 nm through the conversion of ortho-Nitrophenyl-b-galactoside (ONPG) by cytoplasmic β-galactosidase. Outer membrane damage was evaluated at 405 nm through the conversion of the chromogenic cephalosporin CENTA by periplasmic β-lactamase.

Permeabilization assay: The permeabilization curves of NCP-2 and NCP-3 are shown in Fig 9. NCP-2 showed the ability to penetrate the outer membrane without destruction of the external membrane itself, performing a destructive action against the inner membrane, with a linear increase of the optical density over time. On the other hand, NCP-3 showed a more pronounced lithic action on both the inner and outer membranes, as suggested by the time-dependent higher increase of the optical density.

### c) Cytotoxicity assessment

**Fig 10:**
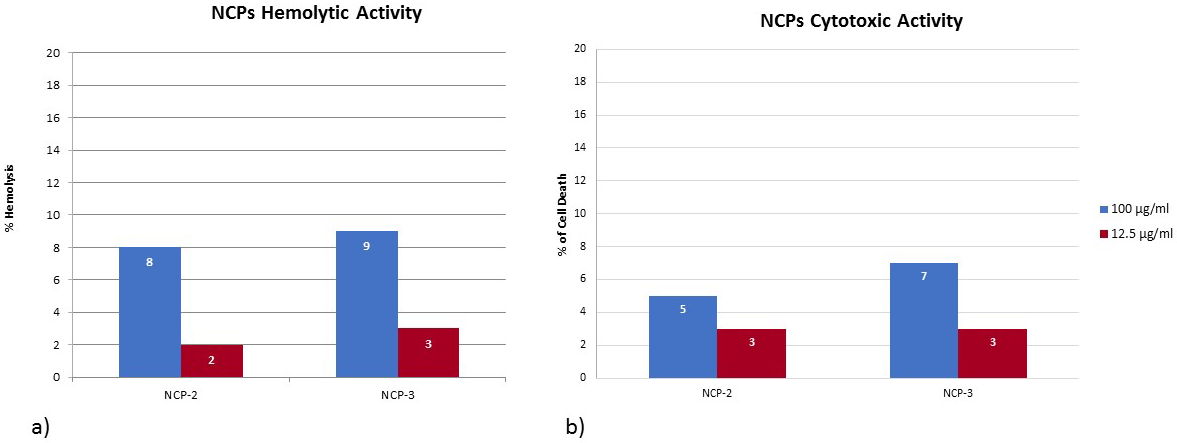
Haemolysis and cytotoxicity tests. (a) Hemolysis assay of NCP-2 and NCP-3 on sheep red blood cells. (b) Cytotoxicity assay of NCP-2 and NCP-3 on Madin-Darby Bovine Kidney (MDBK) cells. Reported percentages are referred to the hemolytic/cytopathic effect scale, where 0% = all intact cells and 100% = hemolytic/cytopathic effect extended to all cells.

Haemolysis test: The haemolytic activity of NCP-2 and NCP-3 is shown in Fig 10A. Both peptides caused an haemolysis lower than 10% at the concentration of 100 μg/ml.

Cytotoxicity test: The cytotoxic activity of NCP-2 and NCP-3 against MDBK cells is shown in Fig 10B. Both peptides caused a cells death lower than 10% at 100 μg/ml.

## Discussion and conclusions

The global onset of antibiotic-resistance in many pathogenic microorganisms is one of the hardest challenges that modern medicine will have to face in the next future. Antibiotic-resistant microorganisms represent a public health threat and are often cause of increased mortality, morbidity and prolonged hospitalization, resulting in huge economic losses all over the world [1,2,5]. Nevertheless, in recent years, pharmaceutical industry has reduced research efforts aimed to discovering new molecules [6]. One of the most promising class of antimicrobials alternative to antibiotics is represented by AMPs. In nature, these molecules are detectable in almost all living organisms and play a key role in the innate immunity response, showing both immuno-modulating and non-specific direct activity against a wide range of microorganisms [9,13]. The target of many cationic peptides is bacterial cytoplasmic membrane, and its depolarization by peptides leads to the destruction of the gradient of the electric potential with consequent cell death, probably due to loss of membrane integrity [8,10,42].

Toxins found in venoms are the result of recruitment events in which an ordinary gene, often encoding for a secreted protein, is duplicated and selectively expressed in the venom gland. Some genes included into the large family of genes encoding for AMPs, are among those which undergone to this process. The subsequent intensive selective pressure on these genes has brought to toxins development, which share many common features, such as the three-dimensional structure, with their encoded proteic ancestors [15]. In addition to their constitutive toxic activities, many AMPs-derived toxins retain some of the original antimicrobial activity [14]. Furthermore, even not AMPs-derived toxins showed antimicrobial activity, as in the case of elapid snakes cardiotoxins (CTXs) that interact with phospholipid membranes similarly to AMPs [16,18,20].

In this study, a new family of antimicrobial peptides has been designed and developed starting from the *Naja atra* cardiotoxin-1 (CTX-1) sequence, using an *in-silico* approach. The first peptide sequence, with the best predictive indexes, has been mainly obtained including residues from the entire first loop and the subsequent β-stranded stretch. This is in agreement with the fact that toxins belonging to the S-type cardiotoxins group, as CTX-1, have the ability to bind phospholipid membranes only with their first loop, through an AMPs-like mechanism [18,19]. Previous studies suggested that bactericidal activity of cationic peptides takes place through the binding of the anionic elements on pathogens outer membrane by electrostatic interactions, followed by the penetration into the membrane by hydrophobic interactions [8–10]. In particular, bulkiest hydrophobic residues, as Trp and Phe, are known to have a role in anchoring a peptide to a membrane bilayer [43,44]. Amphiphilic conformation enhance bactericidal activity, which is undertaken through bacterial membranes destruction [21]. In order to increase the antimicrobial activity of the developed peptides, sequences have been designed to have a higher positive charge density and a more balanced hydrophilic and hydrophobic surfaces ratio than the original stretch. Through CD spectra analyses, we assessed the structural conformation of these peptides. In aqueous environment, NCPs showed a random coil structure, as it is often observed for small peptides and in particular for AMP peptides [21]. In fact, in aqueous solution many antimicrobial peptides are unstructured and fold into their final conformation upon partitioning into biological membranes [28,45]. In agreement with what observed for the NCP3 spectrum in buffer, it was reported that beta-structured antimicrobial peptides are typically more ordered also in aqueous solution [28]. In presence of liposomes wide differences were showed among the secondary structure of the two main sequences, NCP-2 and NCP-3 (Fig 4b). NCP-2 possesses two proline residues: one near the N-terminus and the other in the center of the polypeptide chain. Proline is known to give rigidity and a bend to the polypeptide chain when placed in the first turn of a helix. On the contrary, proline causes kinks in helices when placed in an inner position. Proline often occurs also in the center of beta turns, inverting the chain direction, and are frequent in structures like beta hairpin, two antiparallel beta-strands connected by a short loop [46]. In NCP-2, the proline near the N-terminus could induce the helical conformation, at least in the first part of the backbone, and the second one in the center could curve the backbone. In NCP-3, the first proline was mutated in tryptophan, so failing in the helix induction. The second central proline is still present and could bend the extended chain in a beta-hairpin conformation. This supersecondary structure, that fit the CD spectrum of NCP-3, is known to occur in membrane beta-AMPs, like protegrin, often in a dimeric form [47]. It is noteworthy that bulky hydrophobic residues with aromatic rings are placed symmetrically with respect to the central proline, i.e. Trp4 on one side and Phe17 and Tyr18 on the other side. This could favor both an intra- or an inter-molecular aromatic stacking, implicating the beta-hairpin or the dimer formation. The beta-peptide oligomerization creates a pore in the membrane, disrupting its integrity. This is in agreement with observed results in bacterial membranes permeation assay (Fig 9). NCP-3 is able to exert a lithic action both on the inner and on the outer bacterial membranes. In some cases AMPs, especially if they adopt a helical conformation, can cross the membrane without pore formation or leakage out of the membrane [28]. These observations are in agreement with the behavior of NCP-2, which showed a helical conformation (Fig 4b). NCP-2 seems to penetrate the outer bacterial membrane without destroying it, but damaging the inner membrane (Fig 9).

Antimicrobial activity assays showed a wide and diversified spectrum of antimicrobial activity. While the effects of NCP-0 against almost all tested bacteria were limited, NCP-2 and NCP-3 showed a broad spectrum of antimicrobial activity. Of particular interest is the good activity against *Pseudomonas aeruginosa* and *Acinetobacter baumannii*. These data are encouraging because the treatment of infections caused by these pathogens is often complicated by their ability to develop multi-drug resistance, both in domestic animals [48,49] and humans [50]. NCP-3a and NCP-3b mutants differ from the NCP-3 only for a single residue at position 4. The presence of an aromatic amino acid at position 4 does not seem to be so relevant for the antimicrobial activity against Gram-negative bacteria as for Gram-positive bacteria (Fig 5). The importance of the role played by aromatic residues, in particular tryptophan and phenylalanine, in determining the affinity for phospholipid membranes and therefore the antimicrobial activity of peptides, was already reported [43]. This finding is confirmed by the good activity showed by NCP-3 even against mycobacteria (Fig 7), fungi (Table 3) and the enveloped virus BoHV-1. Actually, the virus envelope is derived from portions of the host cell membranes, rich in phospholipids and proteins, thus representing a potential target for NCPs action. One of the great advantages of AMPs, compared to conventional antimicrobial molecules, is the high speed of action [51]. NCPs showed bactericidal activity within 5–60 min of contact for most of the bacteria tested in the time-kill assay (Fig 6). These results were confirmed by the propidium iodide assay, that showed the damage of *Pseudomonas aeruginosa* inner membrane within 15 min of contact with NCP-2 or NCP-3 (Fig 8).

To avoid environmental interferences, in the first part of NCPs evaluation each test was carried out under optimal conditions, i.e. in PB, for NCPs activity. To mimic *in vivo* conditions, tests were then performed in more complex media (salt rich media and Mueller-Hinton medium). The activity of NCP-2 and especially that of NCP-3 seems to be very little affected by the presence of NaCl. In presence of 20% MH broth, NCP-3 maintained a good antimicrobial activity against tested Gram-negative bacteria, while the activity against MRSA was slightly reduced. An environment with high salt concentrations, as well as the presence of a complex mixture of salts and peptones (Mueller-Hinton broth), weakens the initial electrostatic interactions between peptides and bacterial cells, reducing their bactericidal activity [52]. A higher positive charge possessed by some AMPs is considered a key feature in maintaining antibacterial activity in presence of salts [52]. Despite their positive charge is not high as other known AMPs, NCP-3 and partly NCP-2 maintained their activity even in presence of high concentrations of sodium chloride or Mueller-Hinton Broth medium. Structural distribution of positive charges could be a key factor for the salt sensitivity of NCP-3 and NCP-2. The particular spacing of the four Lys residues gives a well delimited surface region with a high basic electrostatic potential. NCPs antimicrobial activity could be enhanced in salts rich environments by adding chelating agents. Since EDTA has showed a synergic effect with AMPs (30), it was used during the anti-mycobacterial activity assay at sub-MIC concentrations. The resazurin assay showed that NCP-3 was able to inhibit *Mycobacterium fortuitum* and *Mycobacterium smegmatis* growth in presence and in absence of EDTA (Fig 7). Growth inhibition was complete except in the case of *Mycobacterium fortuitum* in absence of EDTA, where a limited growth was observed.

Many AMPs, including those present in venoms, showed cytotoxicity on eukaryotic cells [53]. Cytotoxicity is a negative feature of AMPs, in view of their *in vivo* use. Therefore, we evaluated *in vitro* the hemolytic and cytotoxic activity of NCP-2 and NCP-3 on sheep RBCs and on MDBK cells. This step is particularly important considering that CTX-1, the protein from which NCPs are derived, is highly cytotoxic. NCP-2 and NCP-3 cytotoxicity was low (Fig 10). In agreement with these results, the two peptides in the presence of neutral DMPC-Chol vesicles mimicking eukaryotic membrane, are not able to assume the ordered secondary structure observed in bacterial mimetic vesicles. In some studies it was investigated the correlation between the extent of ordered secondary structure induced in an AMPs in the presence of membrane and its influence on their hemolytic and antibacterial properties [54 and references therein]. It is believed that the electrostatic differences in the composition of bacterial and mammalian plasma membranes (anionic versus neutral) are responsible for selective toxicity of AMPs [55]. These different types of membranes do induce different peptide structures. It was found that AMP peptides possess a higher secondary structure content in the presence of anionic vesicles and not of zwitterionic ones. In addition, mutations that reduce the ordered secondary structure amount cause reduced antibacterial and hemolytic properties. Vice-versa, peptides that retain high helical content in presence of zwitterionic lipids tend to be more hemolytic [56–58]. For NCP-3, the smaller amount of beta structure present in DMPC-Chol with respect to DMPE-DMPG vesicles likely is not enough to recreate the complete beta-hairpin structure that is able to start an oligomerization forming a destructive pore. On the contrary, NCP-0 is able to assume, in the presence of neutral vesicles, an ordered structure that could indicate an interaction with the membrane. This is in agreement with the fact that NCP-0 sequence is more similar to that of CTX-1, whose cytotoxic activity on eukaryotic cells is reported in literature [17], compared to NCP-2 and NCP-3 sequences.

However, *in vitro* results may not depict the *in vivo* behavior. Therefore, despite the encouraging results, the use of these peptides in the treatment of infectious disease still require further studies in order to overcome some issues common to many AMPs. The *in vivo* use of antimicrobial peptides is limited due to the loss of functionality in serum, owing to enzymatic degradation and binding to serum components. Moreover, there are still few data available regarding the *in vivo* toxicities of AMPs and the stability of the peptide/peptide-formulation [13].

The generation of AMPs with new features by proteolytic cleavage of known proteins is a widespread practice [59]. However, the design of peptides with antimicrobial activity starting from the sequence of a toxin not related to AMPs is an innovative approach. Even the use of highly cytotoxic toxins, such as CTX-1, should not be excluded, because the derived peptides, through subsequent modifications of their amino acid sequences, could lost unwanted features, as cytotoxicity, but maintaining antimicrobial activity. Furthermore, we observed that in NCP-3 a single amino acid residue substitution, caused deep structural changes and increased the antimicrobial activity against Gram negative and Gram positive bacteria.

Considering our results, the approach here reported could represent a new and promising way for the discovery and development of new antimicrobial peptides for the treatment of infectious diseases.
