# Correction: Novel *Naja atra* cardiotoxin 1 (CTX-1) derived antimicrobial peptides with broad spectrum activity

**DOI:** 10.1371/journal.pone.0196295

**Published:** 2018-04-18

**Authors:** The PLOS ONE Staff

ln Table 1, the sequences of NCP-2, NCP-3, NCP-3a and NCP-3b are incorrect. Specifically, the glycine (G) residue in position 13 is incorrectly substituted with isoleucine (I). Additionally, the substitutions in these sequences are not formatted correctly. They should be marked in bold and underlined. The publisher apologizes for this error. Please see the corrected Table 1 here.

**Table 1:** Amino acidic sequences of peptides belonging to the NCPs family.

Sequence ID	Primary Structure
Original CTX-1 stretch	KLIPIASKTCPAGKNLCYKM
NCP-0	KLIPIASKTCPAGKNLCYK**I**
NCP-2	KLIPI**L**SKT**I**PA**I**KNL**F**YK**I**
NCP-3	KLI**W**I**L**SKT**I**PA**I**KNL**F**YK**I**
NCP-3a	KLI**F**I**L**SKT**I**PA**I**KNL**F**YK**I**
NCP-3b	KLI**L**I**L**SKT**I**PA**I**KNL**F**YK**I**

The substitutions in the amino acids composition, compared to the Original CTX-1 stretch, are marked in bold and underlined.

## References

[1] Sala A, Cabassi CS, Santospirito D, Polverini E, Flisi S, Cavirani S, et al (2018) Novel *Naja atra* cardiotoxin 1 (CTX-1) derived antimicrobial peptides with broad spectrum activity. PLoS ONE 13(1): e0190778 https://doi.org/10.1371/journal.pone.0190778 29364903 10.1371/journal.pone.0190778 PMC5783354

